# Supplementary material for: What Factors Shape the Flyability in Bats?—The Perspective from Bat’s Wing Development
Source: Biology (Basel). 2025 Oct 30;14(11):1524. doi: 10.3390/biology14111524 (PMC12650304; doi:10.3390/biology14111524)
Supplement: Supplementary file 1 [file biology-14-01524-s001.zip › biology-3882149-supplementary.pdf]

Supplementary information

Table S1. Summary of studies retrieved to date using the keyword "bat flight" in the Web of Science (WOS) database (up to 2025). This table compiles most studies associated with "bat flight," presenting their study species, examples, research objectives, methods, conclusions, and thematic categories (e.g., aerodynamics and kinematics, energetics and metabolism). Although this keyword may not cover the full scope of bat flight research, the table serves as a valuable reference for future studies in this field.

Species	Examples	Objectives	Method	Conclusion	Thematic category
Carollia perspicillata	[1]	Test whether intraspecific wing morphology predicts flight energetics	Controlled free-flight, post-flight infrared thermography, and biophysical heat-loss modeling	Wing morphology explains part of energy cost and reveals performance trade-offs and sexual dimorphism	Aerodynamics and kinematics
Balionycteris maculata<br>*Cynopterus brachyotis*<br>*Eonycteris spelaea*	[2]	Test whether vertical habitat use is partitioned by wing morphology	Height-stratified mist-netting (1–30 m), wing morphometrics (wingspan, wing area, mass), AR & WL computation, Kruskal–Wallis and ANOVA–GT2	Height groups match morphology; low WL/AR occupy clutter; small morphological differences structure vertical niches	
Rhinolophus marshalli	[3]	Describe echolocation calls and wing morphology, including sex variation	Field mist-netting; wing morphometrics; AR/WL computation; time-expanded call analyses	CF–FM calls and low WL/AR documented; sex differences weak; baseline data provided	
Tadarida brasiliensis	[4]	Quantify speed-dependent kinematics and wake aerodynamics; compare with frugivorous bats and swifts	Wind-tunnel flights; synchronized high-speed video + time-resolved PIV; 3D marker-based kinematics; vortex circulation analysis	Wake is swift-like (tip-vortex peak mid-downstroke; low upstroke lift); root vortex weak/absent at high speed	
69 species from 7 families	[5]	Test whether wing morphology predicts climatic niche position via dispersal ability	Geometric morphometrics; PCA and phylogenetically aligned PCA; PGLS; PLS; ancestral-state reconstruction	Wing shape and size vary with guilds and covary with flight descriptors, showing convergent ecomorphs across familie	
Phoniscus papuensis	[6]	Test if wing morphology matches flight behaviour and habitat preference	Wing morphometrics; obstacle-array flight tests; comparative metrics	Phoniscus papuensis shows lower wing loading and higher success through obstacles, indicating clutter-adapted slow agile flight	
152 species from 15 families	[7]	Test correlated evolution between wing morphology and echolocation under phylogeny	Compile RWL and AR, call duration and peak frequency, foraging guilds; log transform; assess phylogenetic signal; PANOVA; PGLS with AICc	Higher RWL and AR predict lower peak frequency; excluding narrow-space flutter detectors, longer duration; guilds predict morphology and calls	

Table S1. Cont.

Species	Examples	Objectives	Method	Conclusion	Thematic category
Phyllostomus hastatus	[8]	Quantify postnatal wing development and flight onset, and assess seasonal wing loading	Field morphometrics of wings and mass; aerodynamic estimates of speed power and transport cost	Young flew by week seven and had minimal wing loading near weaning, morphology reduces energy and improves maneuverability, adult wing loading varies seasonally	Aerodynamics and kinematics
51 species from 7 families	[9]	Characterize wing shape and test links to habitat use and guilds	igital wing morphometrics; aspect ratio and wing loading and relative wing loading; guild classification; morphospace and ANOVA	Wing shape predicts flight pattern and habitat use; high AR and RWL indicate fast open-space flight; low AR and RWL indicate slow manoeuvrable flight; differences among guilds are significant	
126 species from Vespertilionidae	[10]	Test whether wing morphology predicts geographic range size	Phylogenetic regressions of range area and longitudinal extent on relative wing loading and aspect ratio; control migration and realm	Range size increases with relative wing loading and aspect ratio; dispersal capacity helps shape distributions	
Passer domesticus<br>*Pycnonotus xanthopygos*<br>*Acridotheres tristis*<br>*Columba livia*<br>*Rhinopoma microphyllum*<br>*Eptesicus fuscus*<br>*Rousettus aegyptiacus*	[11]	Link wingbeat kinematics to acoustic impulse strength and frequency	Synchronized motion capture and calibrated microphones; single-wing and moving-surface tests; wavelet analysis	Sound pressure level scales with wing area; bats are quieter than birds; downstroke yields negative pressure then positive at reversal; phase depends on angle; curvature raises frequency	
Cynopterus sphinx	[12]	Track postnatal wing growth and model flight performance across development	Captive cohort, 5-day morphometrics and flight tests, logistic growth fits, aerodynamic models for power, speed, turning radius	Flutter at 40 days, sustained flight at 55 days, wing loading minimum near 35 days then increases, speeds dip then rise, logistic fits best	
Miniopterus schreibersii	[13]	Propose a wingtip fold mechanism that aids thrust at slow flight	High-speed video of take-off wingstrokes, qualitative kinematic analysis	Wingtip folds downward late in upstroke then straightens before downstroke, likely adding thrust for slow manoeuvres	
19 species from Vespertilionidae<br>*Rhinolophus ferrumequinum*<br>*Rhinolophus hipposideros*<br>*Miniopterus schreibersii*<br>*Tadarida teniotis*	[14]	Test whether echolocation parameters and wing morphology predict vertical flight height across a bat community	Two-microphone arrays on 48 wind masts in France and Belgium for over 8,000 nights; time-of-arrival call localization; acoustic ID with manual checks; AR and WL from skeletal measures; traits linked to height with GLMM and Kendall correlations	Peak frequency and bandwidth predict the proportion of flights at height; higher aspect ratio and higher wing loading associate with higher flight heights; call duration is a weaker predictor with *Rhinolophus* as outliers	

Table S1. Cont.

Species	Examples	Objectives	Method	Conclusion	Thematic category
Carollia perspicillata	[15]	Report disproportional dwarfism, infer flight aerodynamic consequences	Skeletal morphometrics versus reference series; estimate wing loading and power–speed from wing metrics and mass	Short wings with normal mass imply higher wing loading, higher minimum power speed, shorter range; adult good condition suggests functional compensation	Aerodynamics and kinematics
Carollia perspicillata	[16]	Compare wing morphology and infer biomechanical and energetic consequences	Wingspan and area; patagium partition; humeral radiographs; AR and WL; allometry and t tests	*T. brasiliensis* shows high AR and WL for fast economical flight; *M. chiloensis* shows low AR and WL with large uropatagium and low humeral I for slow maneuverable flight; wing form reflects trade offs	
Myotis chiloensis					
Rhinolophus hipposideros	[17]	Quantify interspecific and intraspecific wing measurement variation, test sex and geographic effects, assess discriminant identification	Measure FA, D5, D3, P4.1, P4.2 on 3081 adults from Bulgaria, Greece, Turkey; GLM, ANOVA, discriminant analysis with FA and P4.1	Species differ in size and shape; females larger; *R. hipposideros* has very short hand wings favoring maneuvering; *R. ferrumequinum* and *R. mehelyi* have longer hand wings favoring faster commuting; two parameter discriminant assigns 98 percent correctly	
Rhinolophus blasii					
Rhinolophus euryale					
Rhinolophus mehelyi					
Rhinolophus ferrumequinum					
Rousettus leschenaultii	[18]	Track postnatal wing growth and flight performance across development	Captive cohort 5–150 days; repeated wing tracings and morphometrics; AR and WL; logistic and Gompertz fits; flight tests every 5 days; aerodynamic models for power, speed, turning radius	Flutter at 45 days; sustained flight at 60 days; independent foraging at 75 days; wingspan fits logistic; wing area fits Gompertz; AR stabilizes by two months; WL decreases to 50 days then increases; power rises after 30 days; Vmp and Vmr drop to 40 days then increase; turning radius minimal at 40–50 days; by 150 days power ≈65 percent and speeds ≈93 percent of postpartum females	
Glossophaga soricina	[19]	Demonstrate unsteady lift in hovering bats and test for a leading-edge vortex	Wind-tunnel free hovering, airflow measured above the wing and analyzed	A leading-edge vortex forms and stays attached during downstroke, enabling sufficient hover lift; this is the first direct proof of unsteady aerodynamics in a hovering vertebrate	

Table S1. Cont.

Species	Examples	Objectives	Method	Conclusion	Thematic category
Pteropus tonganus<br>*Pteropus samoensis*	[20]	Compare wing morphology, flight behaviour, and habitat use to test ecological overlap under similar morphology	Mist netting with wing tracings to compute span, area, aspect ratio, wing loading; radio telemetry of commuting; diurnal soaring observations and video; t tests	Adults are morphologically similar with intermediate aspect ratio and wing loading; *P. samoensis* soars diurnally on thermals and slope updrafts; *P. tonganus* commutes long nocturnal distances; shared load carrying likely drives convergent intermediate wing loading	Aerodynamics and kinematics
Cynopterus sphinx	[21]	Functional reconstruction of shoulder girdle motion in flying bats	Morphological dissection, X-ray fluoroscopy, and high-speed videography	Wing motion is driven mainly by humerus movement; shoulder girdle acts as a crank-like mechanism with minor kinematic contribution	
Cynopterus brachyotis	[22]	Evaluate the inertial energy costs of wing folding during bat flight	3D wing kinematics and inertial modeling across species	Wing folding reduces total inertial cost but increases upstroke energy use	
Cynopterus brachyotis	[23]	Assess individual kinematic strategies in response to increased body mass	3D wing kinematics reconstruction in wind tunnel and flight corridor under controlled loading	Bats exhibit individual-specific kinematic plasticity to generate lift under load	
Cynopterus brachyotis	[24]	Quantify wake structure and kinematic variation in bat flight	Time-resolved particle image velocimetry and high-speed 3D kinematic reconstruction	Bats produce repeatable vortex patterns with individual-specific kinematic strategies	
Cynopterus brachyotis	[25]	Quantify the dynamic wake structure of bat flight	Synchronized high-speed kinematics and time-resolved PIV	Bats generate closed-loop wake vortices with lift throughout the wingbeat	
Cynopterus brachyotis	[26]	Aerodynamic analysis of bat flight symmetry	Wind tunnel motion capture and airflow simulation	Symmetric modeling reveals biomechanical consistency	
Cynopterus brachyotis	[27]	Quantifying dimensional complexity in bat flight	Wind tunnel kinematic tracking with proper orthogonal decomposition	Bat flight complexity is high but can be simplified via joint groupings	
Cynopterus brachyotis<br>*Rousettus aegyptiacus*<br>*Plecotus auritus*<br>*Myotis daubentonii*	[28]	Comparative analysis of bat flight biomechanics	Wind tunnel experiments and aerodynamic modeling	Flight efficiency shaped by wing compliance and sensory control	
Myotis daubentonii	[29]	Energy-saving mechanisms in ground-effect flight	Wind tunnel tests with tomographic particle image velocimetry	Ground effect reduces bat flight power by 29%	
Myotis izecksohni<br>*Lasiurus ega*	[30]	Predict bat takeoff performance using morphology	Morphometrics and flight acceleration testing with GLM modeling	Larger mass and higher aspect ratio predict greater initial acceleration	
Myotis lucifugus<br>*Myotis ciliolabrum*<br>*Myotis evotis*	[31]	Determine environmental predictors and energetic costs of winter bat flight	Acoustic monitoring and species-specific bioenergetic modeling	Temperature and wind govern bat activity; thermogenesis offsets flight cost	

Table S1. Cont.

Species	Examples	Objectives	Method	Conclusion	Thematic category
Myotis velifer<br>*Tadarida brasiliensis*	[32]	Comparative analysis of wake structure and flight kinematics in aerial-hawking bats	Wind tunnel-based particle image velocimetry and high-speed 3D videography	Species-specific morphological traits influence wake architecture and aerodynamic efficiency, especially at low speeds	
Eptesicus fuscus	[33]	Coupling of echolocation behavior and flight dynamics during obstacle navigation	Synchronized high-speed motion tracking and audio recording in flight corridor task	Bats tightly coordinate sonar call timing with wingbeat phase and adapt signals to maneuvering demands	
Eptesicus fuscus	[34]	Fine-scale analysis of bat responses to forest structure	Integration of thermal infrared imaging and terrestrial LiDAR scanning	Bats exhibit individual, obstacle-avoiding flight paths shaped by forest clutter	
Tadarida brasiliensis	[35]	Evaluate aerodynamic costs and benefits of large external ears in bat flight	Wind tunnel PIV and mechanical power modeling	Large ears increase both lift and drag, raising power cost and favoring slow flight	
74 species from Phyllostomidae	[36]	Uncover unsteady aerodynamic mechanisms in bat hovering flight	Wind tunnel particle image velocimetry on live bats	Bats generate stable leading edge vortices to enhance lift	
Glossophaga soricina	[37]	Characterize wake structures in bat flapping flight	Wind tunnel experiments with digital particle image velocimetry (DPIV)	Bat wakes are more complex than bird models and include active lift in upstroke	
Glossophaga soricina<br>*Leptonycteris yerbabuenae*	[38]	Comprehensive evaluation of bat flight mechanics	Particle image velocimetry and kinematic analysis	Lift and thrust shaped by wing flexibility and unsteady aerodynamics	Aerodynamics and kinematics
Glossophaga soricina	[39]	Optimize bat wing efficiency through motion and structure	Fluid–structure simulations of parameterized bat-like wings	Fibre-reinforced membranes delay flutter and enhance efficiency	
Leptonycteris yerbabuenae	[40]	Kinematic and aerodynamic comparison of nectar-feeding bats	Wind tunnel testing with high-speed stereo filming and DPIV	Flight mechanics are similar despite body size difference	
Leptonycteris yerbabuenae	[41]	Control of leading edge vortex via stroke plane modulation	Wind tunnel tests using a bat-inspired robotic flapper	Stroke plane angle modulates LEV strength and stability	
Carollia perspicillata	[42]	Determine the role of tendon elasticity in bat wing muscle mechanics	XROMM and fluoromicrometry during ascending flight	Elastic tendons reduce muscle strain and recycle energy in flight	
Carollia perspicillata<br>*Desmodus rotundus*<br>*Sturnira lilium*<br>*Artibeus lituratus*	[30]	Predict bat takeoff performance using morphology	Morphometrics and flight acceleration testing with GLM modeling	Larger mass and higher aspect ratio predict greater initial acceleration	
Hipposideros pratti	[43]	Computational analysis of aerodynamic forces in bat forward flight	High-resolution motion capture and immersed boundary flow simulation	Lift is enhanced during upstroke by wing rotation, with efficient unsteady aerodynamics	
Hipposideros armiger	[44]	Automated design of bat-inspired joint geometry	Motion capture and empirical potential-based kinematic modeling	Learned joint shapes restrict motion to biologically observed configurations	

Table S1. Cont.

Species	Examples	Objectives	Method	Conclusion	Thematic category
Pteronotus personatus; *Rhinopoma microphyllum*; *Tadarida brasiliensis*	[28]	Comparative analysis of bat flight biomechanics	Wind tunnel experiments and aerodynamic modeling	Flight efficiency shaped by wing compliance and sensory control	Aerodynamics and kinematics
Molossus rufus	[30]	Predict bat takeoff performance using morphology	Morphometrics and flight acceleration testing with GLM modeling	Larger mass and higher aspect ratio predict greater initial acceleration	
Thyroptera tricolor	[45]	Link roosting ecology to landing biomechanics in bats	High-speed videography and force-plate analysis in artificial leaf setup	Unique landing style with high impact forces reflects roost-driven biomechanical specialization	
Miniopterus natalensis	[46]	Wing bone mineralization patterns for flight adaptation	Biomechanical and proteomic profiling	Reduced mineralization enhances wing flexibility	
Mystacina tuberculata	[47]	Assess vertical flight activity in relation to forest structure	Height-stratified acoustic monitoring with vegetation cover analysis	Bat activity peaks at mid-height and declines with increased vegetation clutter	
Carollia perspicillata	[1]	Test whether intraspecific wing morphology predicts flight energetics	Controlled free-flight, post-flight infrared thermography, and biophysical heat-loss modeling	Wing morphology explains part of energy cost and reveals performance trade-offs and sexual dimorphism	Energetics and metabolism
Phyllostomus hastatus	[8]	Quantify postnatal wing development and flight onset, and assess seasonal wing loading	Field morphometrics of wings and mass; aerodynamic estimates of speed power and transport cost	Young flew by week seven and had minimal wing loading near weaning, morphology reduces energy and improves maneuverability, adult wing loading varies seasonally	
Cynopterus sphinx	[12]	Track postnatal wing growth and model flight performance across development	Captive cohort, 5-day morphometrics and flight tests, logistic growth fits, aerodynamic models for power, speed, turning radius	Flutter at 40 days, sustained flight at 55 days, wing loading minimum near 35 days then increases, speeds dip then rise, logistic fits best	
Carollia perspicillata; *Myotis chiloensis*	[16]	Compare wing morphology and infer biomechanical and energetic consequences	Wingspan and area; patagium partition; humeral radiographs; AR and WL; allometry and t tests	*T. brasiliensis* shows high AR and WL for fast economical flight; *M. chiloensis* shows low AR and WL with large uropatagium and low humeral I for slow maneuverable flight; wing form reflects trade offs	

Table S1. Cont.

Species	Examples	Objectives	Method	Conclusion	Thematic category
Rousettus leschenaultii	[18]	Track postnatal wing growth and flight performance across development	Captive cohort 5–150 days; repeated wing tracings and morphometrics; AR and WL; logistic and Gompertz fits; flight tests every 5 days; aerodynamic models for power, speed, turning radius	Flutter at 45 days; sustained flight at 60 days; independent foraging at 75 days; wingspan fits logistic; wing area fits Gompertz; AR stabilizes by two months; WL decreases to 50 days then increases; power rises after 30 days; Vmp and Vmr drop to 40 days then increase; turning radius minimal at 40–50 days; by 150 days power ≈65 percent and speeds ≈93 percent of postpartum females	
Eidolon helvum	[48]	Assess wind-related energetic modulation in free-flying fruit bats	Bio-logging using GPS and tri-axial accelerometry	Headwinds increase ODBA, but airspeed does not	
Myotis lucifugus<br>*Myotis ciliolabrum*<br>*Myotis evotis*<br>*Eptesicus fuscus*	[31]	Determine environmental predictors and energetic costs of winter bat flight	Acoustic monitoring and species-specific bioenergetic modeling	Temperature and wind govern bat activity; thermogenesis offsets flight cost	Energetics and metabolism
Eptesicus fuscus	[34]	Fine-scale analysis of bat responses to forest structure	Integration of thermal infrared imaging and terrestrial LiDAR scanning	Bats exhibit individual, obstacle-avoiding flight paths shaped by forest clutter	
Carollia sowelli	[49]	Quantify the energetic impact of rain on bat flight	^{13}C-bicarbonate metabolic tracer in controlled rain exposure	Wet fur and wing membranes double flight energy cost	
Carollia perspicillata	[50]	Quantify regional muscle temperature gradients during bat flight	Thermocouple-based in vivo muscle temperature measurement in lab and field	Distal wing muscles operate at significantly lower temperatures than the core	
Carollia perspicillata	[42]	Determine the role of tendon elasticity in bat wing muscle mechanics	XROMM and fluoromicrometry during ascending flight	Elastic tendons reduce muscle strain and recycle energy in flight	
Artibeus jamaicensis	[51]	Link muscle fiber histochemistry to motor control strategy	Myofibrillar ATPase and oxidative enzyme histochemical staining	Two fast fibers enable dual-mode muscle control	
69 neotropical species from 7 families	[52]	Assess how wing shape relates to foraging guilds and flight descriptors, and infer ancestral states and convergence	Geometric morphometrics; PCA and phylogenetically aligned PCA; PGLS; PLS; ancestral-state reconstruction	Wing shape and size vary with guilds and covary with flight descriptors, showing convergent ecomorphs across families	
11 species from Phyllostomidae	[53]	Link wing shape to diet specialization and plant–bat network position	Wing geometric morphometrics in a phylogenetic context; fecal seed diets; vegetation plots; bipartite network metrics; AR and WL with GLMs	Wide short wings generalist and less nested; elongated pointed wings specialist and more nested; aspect ratio best predicts subcanopy use; wing size shows stronger phylogenetic signal than shape	Morphology and development

Table S1. Cont.

Species	Examples	Objectives	Method	Conclusion	Thematic category
Myotis tricolor	[54]	Test if *M. tricolor* can glean and relate performance to wing shape and echolocation	Flight-room foraging trials, wing morphology metrics, echolocation analyses, discriminant statistics	Species seldom gleans; pointed wing tips and narrow bandwidth calls without harmonics may constrain gleaning	Morphology and development
Carollia perspicillata	[15]	Report disproportional dwarfism, infer flight aerodynamic consequences	Skeletal morphometrics versus reference series; estimate wing loading and power–speed from wing metrics and mass	Short wings with normal mass imply higher wing loading, higher minimum power speed, shorter range; adult good condition suggests functional compensation	
Rhinolophus hipposideros<br>*Rhinolophus blasii*<br>*Rhinolophus euryale*<br>*Rhinolophus mehelyi*<br>*Rhinolophus ferrumequinum*	[17]	Quantify interspecific and intraspecific wing measurement variation, test sex and geographic effects, assess discriminant identification	Measure FA, D5, D3, P4.1, P4.2 on 3081 adults from Bulgaria, Greece, Turkey; GLM, ANOVA, discriminant analysis with FA and P4.1	Species differ in size and shape; females larger; *R. hipposideros* has very short hand wings favoring maneuvering; *R. ferrumequinum* and *R. mehelyi* have longer hand wings favoring faster commuting; two parameter discriminant assigns 98 percent correctly	
Rousettus leschenaultii	[18]	Track postnatal wing growth and flight performance across development	Captive cohort 5–150 days; repeated wing tracings and morphometrics; AR and WL; logistic and Gompertz fits; flight tests every 5 days; aerodynamic models for power, speed, turning radius	Flutter at 45 days; sustained flight at 60 days; independent foraging at 75 days; wingspan fits logistic; wing area fits Gompertz; AR stabilizes by two months; WL decreases to 50 days then increases; power rises after 30 days; Vmp and Vmr drop to 40 days then increase; turning radius minimal at 40–50 days; by 150 days power ≈65 percent and speeds ≈93 percent of postpartum females	
Pteronotus mesoamericanus	[55]	Test whether individual wing shape predicts dietary specialization and diet dissimilarity	Guano DNA metabarcoding; geometric morphometrics with fourteen landmarks; PCA; network nestedness and Bray–Curtis PCoA; multiple regressions	Rounded broad wings indicate generalist diets with lower nestedness; pointed triangular wings indicate specialist diets with higher nestedness; wing shape correlates with diet dissimilarity	

Table S1. Cont.

Species	Examples	Objectives	Method	Conclusion	Thematic category
Eptesicus fuscus<br>*Lasiurus borealis*<br>*Nycticeius humeralis*<br>*Corynorhinus rafinesquii*<br>*Lasiurus seminolus*<br>*Myotis austroriparius*	[56]	Quantify trophic niche overlap with stable isotopes; relate niches to wing morphology	Fur ^{13}C and ^{15}N; mist netting; wingspan and area; aspect ratio and wing loading; SIBER ellipses; ANOVA with Tukey; Pearson correlations	Some species show distinct isotope niches while others overlap; aspect ratio increases from gleaners to open space; ^{15}N weakly negatively correlates with aspect ratio; *C. rafinesquii* niche is narrow and *M. austroriparius* broad	Morphology and development
Eptesicus fuscus	[57]	Comparative analysis of capillary geometry in high- and low-aerobic muscles	Morphometric and ultrastructural quantification via electron microscopy	Bat flight muscle shows enhanced capillary-fiber interface for O_2 exchange	
Lasiurus ega<br>*Carollia perspicillata*<br>*Desmodus rotundus*<br>*Sturnira lilium*<br>*Artibeus lituratus*<br>*Molossus rufus*	[30]	Predict bat takeoff performance using morphology	Morphometrics and flight acceleration testing with GLM modeling	Larger mass and higher aspect ratio predict greater initial acceleration	
Carollia perspicillata	[58]	Molecular basis of bat wing morphogenesis	Comparative gene expression and functional enhancer replacement experiments	Gene expression shifts drive wing specialization	
Pteropus poliocephalus<br>*Pteropus hypomelanus*	[59]	Molecular profiling of bat wing development	RNA-seq and ChIP-seq of developing limbs	Gene regulation drives forelimb specialization	
Balionycteris maculata<br>*Cynopterus brachyotis*<br>*Eonycteris spelaea*	[2]	Test whether vertical habitat use is partitioned by wing morphology	Height-stratified mist-netting (1–30 m), wing morphometrics (wingspan, wing area, mass), AR & WL computation, Kruskal–Wallis and ANOVA–GT2	Height groups match morphology; low WL/AR occupy clutter; small morphological differences structure vertical niches	Ecology
69 species from 7 families	[5]	Test whether wing morphology predicts climatic niche position via dispersal ability	Geometric morphometrics; PCA and phylogenetically aligned PCA; PGLS; PLS; ancestral-state reconstruction	Wing shape and size vary with guilds and covary with flight descriptors, showing convergent ecomorphs across familie	
Phoniscus papuensis<br>*Nyctophilus bifax*	[6]	Test if wing morphology matches flight behaviour and habitat preference	Wing morphometrics; obstacle-array flight tests; comparative metrics	Phoniscus papuensis shows lower wing loading and higher success through obstacles, indicating clutter-adapted slow agile flight	
152 species from 15 families	[7]	Test correlated evolution between wing morphology and echolocation under phylogeny	Compile RWL and AR, call duration and peak frequency, foraging guilds; log transform; assess phylogenetic signal; PANOVA; PGLS with AICc	Higher RWL and AR predict lower peak frequency; excluding narrow-space flutter detectors, longer duration; guilds predict morphology and calls	

Table S1. Cont.

Species	Examples	Objectives	Method	Conclusion	Thematic category
Artibeus lituratus<br>*Artibeus planirostris*<br>*Platyrrhinus lineatus*	[60]	Describe fruits used, feeding-roost selection, and seed-dispersal patterns in an urban area	Collected and identified seeds beneath feeding roosts; mist netting near roosts; assessed vegetation and urban variables; estimated dispersal distance	Bats preferred tall darker trees and dispersed seeds on average 105.5 meters including many exotics with implications for urban ecosystems	Ecology
51 species from 7 families	[9]	Characterize wing shape and test links to habitat use and guilds	igital wing morphometrics; aspect ratio and wing loading and relative wing loading; guild classification; morphospace and ANOVA	Wing shape predicts flight pattern and habitat use; high AR and RWL indicate fast open-space flight; low AR and RWL indicate slow manoeuvrable flight; differences among guilds are significant	
19 species from Vespertilionidae<br>*Rhinolophus ferrumequinum*<br>*Rhinolophus hipposideros*<br>*Miniopterus schreibersii*<br>*Tadarida teniotis*	[14]	Test whether echolocation parameters and wing morphology predict vertical flight height across a bat community	Deploy two-microphone arrays on 48 wind masts in France and Belgium for over 8000 nights; localize calls by time differences of arrival; identify species acoustically with manual verification; compute aspect ratio and wing loading from bone measurements; relate traits to height with GLMM and Kendall correlations	Peak frequency and bandwidth predict the proportion of flights at height; higher aspect ratio and higher wing loading associate with higher flight heights; call duration is a weaker predictor with *Rhinolophus* as outliers	
Rhinolophus hipposideros<br>*Rhinolophus blasii*<br>*Rhinolophus euryale*<br>*Rhinolophus mehelyi*<br>*Rhinolophus ferrumequinum*	[17]	Quantify interspecific and intraspecific wing measurement variation, test sex and geographic effects, assess discriminant identification	Measure FA, D5, D3, P4.1, P4.2 on 3081 adults from Bulgaria, Greece, Turkey; GLM, ANOVA, discriminant analysis with FA and P4.1	Species differ in size and shape; females larger; *R. hipposideros* has very short hand wings favoring maneuvering; *R. ferrumequinum* and *R. mehelyi* have longer hand wings favoring faster commuting; two parameter discriminant assigns 98 percent correctly	
Eptesicus fuscus<br>*Lasiurus borealis*<br>*Nycticeius humeralis*<br>*Corynorhinus rafinesquii*<br>*Lasiurus seminolus*<br>*Myotis austroriparius*	[56]	Quantify trophic niche overlap with stable isotopes; relate niches to wing morphology	Fur ^{13}C and ^{15}N; mist netting; wingspan and area; aspect ratio and wing loading; SIBER ellipses; ANOVA with Tukey; Pearson correlations	Some species show distinct isotope niches while others overlap; aspect ratio increases from gleaners to open space; ^{15}N weakly negatively correlates with aspect ratio; *C. rafinesquii* niche is narrow and *M. austroriparius* broad	

Table S1. Cont.

Species	Examples	Objectives	Method	Conclusion	Thematic category
Pteropus tonganus	[20]	Compare wing morphology, flight behaviour, and habitat use to test ecological overlap under similar morphology	Mist netting with wing tracings to compute span, area, aspect ratio, wing loading; radio telemetry of commuting; diurnal soaring observations and video; t tests	Adults are morphologically similar with intermediate aspect ratio and wing loading; *P. samoensis* soars diurnally on thermals and slope updrafts; *P. tonganus* commutes long nocturnal distances; shared load carrying likely drives convergent intermediate wing loading	Ecology
Pteropus samoensis					
Myotis septentrionalis	[61]	Habitat-specific bat activity patterns in forested landscapes	Active acoustic monitoring across diverse habitats	Bat activity peaks over still-water and varies by morphology	
Lasiurus borealis					
Lasiurus cinereus					
Eptesicus fuscus					
Mystacina tuberculata	[46]	Assess vertical flight activity in relation to forest structure	Height-stratified acoustic monitoring with vegetation cover analysis	Bat activity peaks at mid-height and declines with increased vegetation clutter	
Eptesicus fuscus	[62]	Somatotopic mapping of wing in primary somatosensory cortex	Electrophysiological recording with calibrated tactile stimulation	Bat wings show enlarged and reversed cortical representation with high tactile sensitivity	Neuro-sensory Integration

References

1. Carneiro, L.d.O.; Mellado, B.; Nogueira, M.R.; Cruz-Neto, A.P.d.; Monteiro, L.R. Flight performance and wing morphology in the bat *Carollia perspicillata*: Biophysical models and energetics. *Integr. Zool.* **2023**, *18*, 876–890.
2. Hodgkison, R.; Balding, S.T.; Zubaid, A.; Kunz, T.H. Habitat structure, wing morphology, and the vertical stratification of Malaysian fruit bats (Megachiroptera: Pteropodidae). *J. Trop. Ecol.* **2004**, *20*, 667–673.
3. Liu, Y.; Jiang, T.; Berquist, S.; Feng, J. Vocal characters and wing morphology of *Rhinolophus marshalli* from Tiantang Cave, Guangxi Province, China. *Mammalia* **2009**, *73*, 373–376.
4. Hubel, T.Y.; Hristov, N.I.; Swartz, S.M.; Breuer, K.S. Changes in kinematics and aerodynamics over a range of speeds in *Tadarida brasiliensis*, the Brazilian free-tailed bat. *J. R. Soc. Interface* **2012**, *9*, 1120–1130. https://doi.org/10.1098/rsif.2011.0838.
5. Varzinczak, L.H. Understanding the relationship between climatic niches and dispersal through the lens of bat wing morphology. *J. Zool.* **2020**, *312*, 239–247.
6. Rhodes, M.P. Wing morphology and flight behaviour of the golden-tipped bat, *Phoniscus papuensis* (Dobson) (Chiroptera: Vespertilionidae). *Aust. J. Zool.* **1995**, *43*, 657–663.
7. Zou, W.; Liang, H.; Wu, P.; Luo, B.; Zhou, D.; Liu, W.; Wu, J.; Fang, L.; Lei, Y.; Feng, J. Correlated evolution of wing morphology and echolocation calls in bats. *Front. Ecol. Evol.* **2022**, *10*, 1031548.
8. Stern, A.A.; Kunz, T.H.; Bhatt, S.S. Seasonal wing loading and the ontogeny of flight in *Phyllostomus hastatus* (Chiroptera: Phyllostomidae). *J. Mammal.* **1997**, *78*, 1199–1209.
9. Marinello, M.M.; Bernard, E. Wing morphology of Neotropical bats: A quantitative and qualitative analysis with implications for habitat use. *Can. J. Zool.* **2014**, *92*, 141–147.
10. Luo, B.; Santana, S.E.; Pang, Y.; Wang, M.; Xiao, Y.; Feng, J. Wing morphology predicts geographic range size in vespertilionid bats. *Sci. Rep.* **2019**, *9*, 4526.
11. Boonman, A.; Yovel, Y.; Eitan, O. Wing-beat frequency and its acoustics in birds and bats. *Integr. Comp. Biol.* **2020**, *60*, 1080–1090.
12. Elangovan, V.; Yuvana Satya Priya, E.; Raghuram, H.; Marimuthu, G. Wing morphology and flight development in the short-nosed fruit bat *Cynopterus sphinx*. *Zoology* **2007**, *110*, 189–196.
13. Nudds, R. The wingtip fold of the bat *Miniopterus schreibersii*: A novel mechanism for thrust generation during slow-flight? *Comp. Biochem. Physiol. Part A Mol. Integr. Physiol.* **2007**, *146*, S115.
14. Roemer, C.; Coulon, A.; Disca, T.; Bas, Y. Bat sonar and wing morphology predict species vertical niche. *J. Acoust. Soc. Am.* **2019**, *145*, 3242–3251.
15. Carneiro, L.; Mellado, B.; Monteiro, L.R.; Nogueira, M.R. Dwarfism in a Seba's short-tailed bat, *Carollia perspicillata*, with comments on its flight aerodynamics. *Can. J. Zool.* **2023**, *101*, 376–384.
16. Iriarte-Díaz, J.; Novoa, F.F.; Canals, M. Biomechanic consequences of differences in wing morphology between *Tadarida brasiliensis* and *Myotis chiloensis*. *Acta Theriol.* **2002**, *47*, 193–200.
17. Dietz, C.; Dietz, I.; Siemers, B.M. Wing measurement variations in the five European horseshoe bat species (Chiroptera: Rhinolophidae). *J. Mammal.* **2006**, *87*, 1241–1251.
18. Elangovan, V.; Raghuram, H.; Satya Priya, E.Y.; Marimuthu, G. Wing morphology and flight performance in *Rousettus leschenaulti*. *J. Mammal.* **2004**, *85*, 806–812.
19. Muijres, F.; Johansson, L.; Barfield, R.; Wolf, M.; Spedding, G.; Hedenström, A. Leading edge vortices lift in bat flight. *Comp. Biochem. Physiol. Part A Mol. Integr. Physiol.* **2008**, *150*, S66.
20. Richmond, J.Q.; Banack, S.A.; Grant, G.S. Comparative analysis of wing morphology, flight behaviour, and habitat use in flying foxes (Genus: *Pteropus*). *Aust. J. Zool.* **1998**, *46*, 283–289.
21. Panyutina, A.A.; Kuznetsov, A.N.; Korzun, L.P. Kinematics of chiropteran shoulder girdle in flight. *Anat. Rec.* **2013**, *296*, 382–394.
22. Riskin, D.K.; Bergou, A.; Breuer, K.S.; Swartz, S.M. Upstroke wing flexion and the inertial cost of bat flight. *Proc. R. Soc. B Biol. Sci.* **2012**, *279*, 2945–2950.
23. Iriarte-Diaz, J.; Riskin, D.K.; Breuer, K.S.; Swartz, S.M. Kinematic plasticity during fight in fruit bats: Individual variability in response to loading. *PLoS ONE* **2012**, *7*, e36665.
24. Hubel, T.Y.; Riskin, D.K.; Swartz, S.M.; Breuer, K.S. Wake structure and wing kinematics: The flight of the lesser dog-faced fruit bat, *Cynopterus brachyotis*. *J. Exp. Biol.* **2010**, *213*, 3427–3440.
25. Hubel, T.Y.; Hristov, N.I.; Swartz, S.M.; Breuer, K.S. Time-resolved wake structure and kinematics of bat flight. *Exp. Fluids* **2009**, *46*, 933–943.

26. Kosara, R. Symmetric bat flight. *Am. Sci.* **2008**, *96*, 348–349.
27. Riskin, D.K.; Willis, D.J.; Iriarte-Díaz, J.; Hedrick, T.L.; Kostandov, M.; Chen, J.; Laidlaw, D.H.; Breuer, K.S.; Swartz, S.M. Quantifying the complexity of bat wing kinematics. *J. Theor. Biol.* **2008**, *254*, 604–615.
28. Hedenström, A.; Johansson, L.C. Bat flight. *Curr. Biol.* **2015**, *25*, R399–R402.
29. Biewener, A.A. Animal locomotion: Near-ground low-cost flights. *Curr. Biol.* **2018**, *28*, R1348–R1349.
30. Miranda, J.; Marchioro, S.; Santos, F.; Ludwig, L.; Zago, L.; Carvalho, F. Body size and wing shape as predictors of the initial flight acceleration in bats of the Brazilian Atlantic Forest. *Papéis Avulsos Zool.* **2025**, *65*, e202565006.
31. Klüg-Baerwald, B.J.; Gower, L.E.; Lausen, C.L.; Brigham, R.M. Environmental correlates and energetics of winter flight by bats in southern Alberta, Canada. *Can. J. Zool.* **2016**, *94*, 829–836.
32. Hubel, T.Y.; Hristov, N.I.; Swartz, S.M.; Breuer, K.S. Wake structure and kinematics in two insectivorous bats. *Philos. Trans. R. Soc. B Biol. Sci.* **2016**, *371*, 20150385.
33. Falk, B.; Kasnadi, J.; Moss, C.F. Tight coordination of aerial flight maneuvers and sonar call production in insectivorous bats. *J. Exp. Biol.* **2015**, *218*, 3678–3688.
34. Yang, X.; Crystal, S.; Alan, S.; Thomas, K.; Nathan, F.; Margrit, B.; Zheng, W.; Zhuosen, W.; Diane, T.; Darius, C.; et al. Study of bat flight behavior by combining thermal image analysis with a LiDAR forest reconstruction. *Can. J. Remote Sens.* **2013**, *39*, S112–S125.
35. Håkansson, J.; Jakobsen, L.; Hedenström, A.; Johansson, L.C. Body lift, drag and power are relatively higher in large-eared than in small-eared bat species. *J. R. Soc. Interface* **2017**, *14*, 20170455.
36. Dickinson, M. Animal locomotion: A new spin on bat flight. *Curr. Biol.* **2008**, *18*, R468–R470.
37. Hedenström, A.; Johansson, L.C.; Wolf, M.; von Busse, R.; Winter, Y.; Spedding, G.R. Bat flight generates complex aerodynamic tracks. *Science* **2007**, *316*, 894–897.
38. Hedenström, A.; Johansson, L.C. Bat flight: Aerodynamics, kinematics and flight morphology. *J. Exp. Biol.* **2015**, *218*, 653–663.
39. Lauber, M.; Weymouth, G.D.; Limbert, G. Rapid flapping and fibre-reinforced membrane wings are key to high-performance bat flight. *J. R. Soc. Interface* **2023**, *20*, 20230466.
40. Von Busse, R.; Wolf, M.; Johansson, C.; Muijres, F.; Winter, Y.; Hedenström, A. Bat flight—Comparison of kinematics and aerodynamics between two nectar feeding species. *Comp. Biochem. Physiol. Part A Mol. Integr. Physiol.* **2009**, *153*, S121–S122.
41. Koekkoek, G.; Muijres, F.T.; Johansson, L.C.; Stuiver, M.; van Oudheusden, B.W.; Hedenström, A. Stroke plane angle controls leading edge vortex in a bat-inspired flapper. *Comptes Rendus Mécanique* **2012**, *340*, 95–106.
42. Konow, N.; Cheney, J.A.; Roberts, T.J.; Waldman, J.R.S.; Swartz, S.M. Spring or string: Does tendon elastic action influence wing muscle mechanics in bat flight? *Proc. R. Soc. B Biol. Sci.* **2015**, *282*, 20151832.
43. Windes, P.; Fan, X.; Bender, M.; Tafti, D.K.; Müller, R. A computational investigation of lift generation and power expenditure of Pratt's roundleaf bat (*Hipposideros pratti*) in forward flight. *PLoS ONE* **2018**, *13*, e0207613.
44. Bender, M.; Guo, J.; Powell, N.; Kurdila, A.; Müller, R. Learning bioinspired joint geometry from motion capture data of bat flight. *Bioinspiration Biomim.* **2019**, *14*, 036013.
45. Boerma, D.B.; Barrantes, J.P.; Chung, C.; Chaverri, G.; Swartz, S.M. Specialized landing maneuvers in Spix's disk-winged bats (*Thyroptera tricolor*) reveal linkage between roosting ecology and landing biomechanics. *J. Exp. Biol.* **2019**, *222*, jeb204024.
46. Eckalbar, W.L.; Schlebusch, S.A.; Mason, M.K.; Gill, Z.; Parker, A.V.; Booker, B.M.; Nishizaki, S.; Muswamba-Nday, C.; Terhune, E.; Nevonen, K.A.; et al. Transcriptomic and epigenomic characterization of the developing bat wing. *Nat. Genet.* **2016**, *48*, 528–536.
47. Scrimgeour, J.; Molles, L.; Waas, J.R. Vertical variation in flight activity of the lesser short-tailed bat in podocarp and beech forests, Central North Island, New Zealand. *New Zealand J. Ecol.* **2013**, *37*, 193–198.
48. O'Mara, M.T.; Scharf, A.K.; Fahr, J.; Abedi-Lartey, M.; Wikelski, M.; Dechmann, D.K.N.; Safi, K. Overall dynamic body acceleration in straw-colored fruit bats increases in headwinds but not with airspeed. *Front. Ecol. Evol.* **2019**, *7*, 200.
49. Voigt, C.C.; Schneeberger, K.; Voigt-Heucke, S.L.; Lewanzik, D. Rain increases the energy cost of bat flight. *Biol. Lett.* **2011**, *7*, 793–795.
50. Rummel, A.D.; Swartz, S.M.; Marsh, R.L. Warm bodies, cool wings: Regional heterothermy in flying bats. *Biol. Lett.* **2019**, *15*, 20190530.
51. Hermanson, J.W.; Foehring, R.C. Histochemistry of flight muscles in the Jamaican fruit bat, *Artibeus jamaicensis*: Implications for motor control. *J. Morphol.* **1988**, *196*, 353–362.

52. Ospina-Garcés, S.M.; Zamora-Gutierrez, V.; Lara-Delgado, J.M.; Morelos-Martínez, M.; Ávila-Flores, R.; Kurali, A.; Ortega, J.; Selem-Salas, C.I.; MacSwiney G., M.C. The relationship between wing morphology and foraging guilds: Exploring the evolution of wing ecomorphs in bats. *Biol. J. Linn. Soc.* **2024**, *142*, 481–498.
53. García-Herrera, L.V.; Ramírez-Fráncel, L.A.; Guevara, G.; Lim, B.K.; Losada-Prado, S. Wing morphology is related to niche specialization and interaction networks in stenodermatine bats (Chiroptera: Phyllostomidae). *J. Mammal.* **2022**, *104*, 347–360.
54. Stoffberg, S.; Jacobs, D.S. The influence of wing morphology and echolocation on the gleaning ability of the insectivorous bat *Myotis tricolor*. *Can. J. Zool.* **2004**, *82*, 1854–1863.
55. Magalhães de Oliveira, H.F.; Camargo, N.F.; Hemprich-Bennett, D.R.; Rodríguez-Herrera, B.; Rossiter, S.J.; Clare, E.L. Wing morphology predicts individual niche specialization in *Pteronotus mesoamericanus* (Mammalia: Chiroptera). *PLoS ONE* **2020**, *15*, e0232601.
56. Veum, S.A.; Tallon, A.K.; Rush, S.A. Trophic niche partitioning among insectivorous bats using a combination of stable isotope analyses and wing morphology. *Acta Chiropterologica* **2025**, *27*, 39–52.
57. Mathieu-Costello, O.; Szewczak, J.M.; Logemann, R.B.; Agey, P.J. Geometry of blood-tissue exchange in bat flight muscle compared with bat hindlimb and rat soleus muscle. *Am. J. Physiol.-Regul. Integr. Comp. Physiol.* **1992**, *262*, R955–R965.
58. Sears, K.E. Molecular determinants of bat wing development. *Cells Tissues Organs* **2007**, *187*, 6–12.
59. Cleland, T.P.; Wang, Z.; Wang, B.; Picu, C.R.; Vashishth, D. Mechano-chemical regulation of bat wing bones for flight. *J. Mech. Behav. Biomed. Mater.* **2021**, *124*, 104809.
60. da Silveira, M.C.; Silveira, M.; Medeiros, L.S.; Aguiar, L.M.S. The role of feeding roosts in seed dispersal service bats provide in urban areas. *Biotropica* **2024**, *56*, e13291.
61. Brooks, R.T.; Ford, W.M. Bat activity in a forest landscape of central Massachusetts. *Northeast. Nat.* **2005**, *12*, 447–462.
62. Chadha, M.; Moss, C.F.; Sterbing-D'Angelo, S.J. Organization of the primary somatosensory cortex and wing representation in the big brown bat, *Eptesicus fuscus*. *J. Comp. Physiol. A* **2011**, *197*, 89–96.

Disclaimer/Publisher's Note: The statements, opinions and data contained in all publications are solely those of the individual author(s) and contributor(s) and not of MDPI and/or the editor(s). MDPI and/or the editor(s) disclaim responsibility for any injury to people or property resulting from any ideas, methods, instructions or products referred to in the content.
